# Role of oxygen in wetting of copper nanoparticles on silicon surfaces at elevated temperature

**DOI:** 10.3762/bjnano.8.45

**Published:** 2017-02-13

**Authors:** Tapas Ghosh, Biswarup Satpati

**Affiliations:** 1 Surface Physics and Material Science Division, Saha Institute of Nuclear Physics, HBNI, 1/AF Bidhannagar, Kolkata-700064, India

**Keywords:** copper, cupric oxide, electron diffraction, galvanic displacement reaction, oxidation, surface wetting, transmission electron microscopy

## Abstract

Copper nanoparticles have been deposited on silicon surfaces by a simple galvanic displacement reaction, and rapid thermal annealing has been performed under various atmospheric conditions. In spite of the general tendency of the agglomeration of nanoparticles to lower the surface energy at elevated temperatures, our plan-view and cross-sectional transmission electron microscopy (TEM), energy dispersive X-ray spectroscopy (EDX) and X-ray diffraction (XRD) analysis shows that the thermal oxidation of the copper nanoparticles and formation of cupric oxide (CuO) on silicon surfaces leads to wetting rather than agglomeration. In contrast, agglomeration has been observed when copper nanoparticles were annealed in a nitrogen environment. The lattice transformation from cubic Cu to monoclinic CuO, and hence the change in surface energy of the particles, assists the wetting process. The occurrence of wetting during the oxidation step implies a strong interaction between the oxidized film and the silicon surface.

## Introduction

The transition metal oxide cupric oxide (CuO) is a stable oxide of copper, and due to its diverse applications, immense research on CuO nanostructure fabrication have been carried out. CuO is a p-type semiconductor with a band gap of 1.4 eV [1–2]. Higher conductivity has been observed in CuO as compared to Cu_2_O, although higher carrier mobility has been observed in Cu_2_O [3]. However, the higher stability of CuO makes it more applicable. In the recent years, several CuO nanostructure syntheses and their applications have been reported. Different shaped CuO nanostructures such as nanowires, nanoplatelets, nanorods, and nanoflowers have been employed as the anode material for lithium ion batteries [4–7], and improved performance has also been observed in composite CuO–carbon nanotube and CuO–graphene systems [8–9]. CuO nanostructures find promising application in many other fields such as gas sensing, catalysis, and arsenic (As) removal in water purification [10–12]. Such differently shaped CuO nanostructures have been synthesized following different processes such as hydrothermal, chemical precipitation, electrochemical as well as other methods [4–7,13]. In this context the combination of the galvanic displacement reaction and the rapid thermal annealing process provides a simple and quick route for the synthesis of CuO nanostructures on Si surfaces.

There are several techniques such as physical vapor deposition (PVD), chemical vapor deposition (CVD), electroplating, etc., that can be used to create Cu films. For the PVD and CVD techniques, high vacuum is required, which takes enormous effort and also increases the production cost. The electroplating technique is comparably less costly. In our present work, we have used the simple, time and cost effective galvanic displacement reaction. The galvanic displacement reaction has a high coverage rate, controllability and can easily be performed at room temperature [14–17]. The galvanic displacement reaction is a part of the electroless deposition family. The name “electroless” signifies that no external voltage is required during the deposition. The other members of electroless deposition family are autocatalytic and substrate catalytic processes. The advantage of the galvanic displacement reaction over the other electroless deposition processes is that it requires no reducing agent, which makes the deposition easier and more pure. The galvanic displacement reaction has been employed for reduction of metals with higher reduction potential on various metal as well as semiconductor surfaces [14–19]. Different nanostructures have been fabricated using the galvanic displacement reaction which leads to many practical applications [20–22].

CuO has important applications in solar cells. With a suitable band gap, CuO has a higher theoretical photocurrent density [23]. The practical conversion efficiency for CuO is low, which may be due to its low carrier concentration [24]. Again, the p-type CuO–n-type Si heterojunction is applicable for solar cells due to their similar electron affinity [25]. The reported open circuit voltage for a p-CuO/n-Si heterojunction solar cell is 0.33 V [26] and after improving the crystalline quality and the interface quality between the CuO and the Si, an increased open circuit voltage of 0.509 V and a high photocurrent density of 12 mA/cm^2^ have been achieved [25]. Therefore, it is important to understand the CuO/Si interface properties. We found that the formation of CuO by the thermal oxidation of Cu nanoparticles on Si surfaces leads to wetting, where no such wetting was observed when the Cu nanoparticles were annealed under a nitrogen atmosphere. This implies that the ambient conditions play an important role in the interface formation.

As mentioned, the galvanic displacement reaction is categorized in the electroless family, where the nanoparticle deposition is possible without any external reducing agent [14–16]. The fundamental criterion for such deposition is that it have a net positive electrode potential between the substrate and the deposited element [14–16]. The standard potential for Cu is 0.34 V and for Si is −0.875 V [27]. Thus the net potential between Cu and Si is 1.215 V, which suggests an easy Cu deposition on Si surfaces, however the insoluble oxide layer on the Si surface prevents the metallization. The addition of hydrofluoric acid (HF) into the copper solution removes such oxide layers formed on the Si surface and assists the galvanic displacement reaction. The reaction takes place in the following way:

[1]
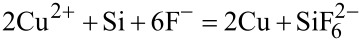


Since SiF_6_^2−^ is water soluble, the galvanic displacement reaction proceeds easily. This dissolution of Si in water from the surface as SiF_6_^2−^ increases the Si surface roughness during the deposition.

In several studies, copper oxide on different substrates have been grown by the direct thermal oxidation of Cu. Pure copper oxide has been easily formed by thermal annealing on indium tin oxide (ITO) or glass [3]; but copper oxidation on a silicon surface may lead to copper silicidation [28]. Papadimitropoulos et al. have observed the formation of copper silicide at low annealing temperatures, and when the temperature is high, pure copper oxide is formed as the oxidation rate overcomes the silicidation [28]. The thermal oxidation produces both the Cu(I) and Cu(II) oxide depending on the duration and temperature of the annealing process. In thermal annealing of copper, Cu(II) oxide is formed at a higher temperature than the Cu(I) oxide [3]. An anealing study of a deposited Cu_2_O film in an oxygen environment also shows the formation of CuO [29]. The general way of formation is Cu to Cu_2_O and then CuO but it is possible to form CuO without formation of any intermediate oxide, by embedding H_2_ [30]. In our present work, we have studied the oxidation of Cu nanoparticles deposited by the galvanic displacement reaction on silicon surfaces at various ambient conditions.

## Experimental

Copper nanoparticles have been deposited on Si(100) substrates by an easy, one-step galvanic displacement reaction. 5 mL of 10 mM CuSO_4_ (Merck, >99%) solution was taken in a small petri dish and 200 μL of HF (48% w/v) was added to the solution. Three TEM-ready (3 mm disc and electron transparent) and one 1 × 1 cm Si substrates were slowly dipped into the solution, kept for 2 min for deposition to occur, then taken out slowly from the solution and dried in air. The Si substrates were prepared for plan-view TEM studies before deposition so that after deposition, the tedious TEM sample preparation procedure (which may affect the loosely bound deposited particles on Si and can change the morphology), was not necessary. To prepare TEM plan-view samples, three discs each of 3 mm diameter were cut from the Si(100) substrate and mechanically thinned to 100 μm, then dimpled and polished to achieve a thickness of approximately 30 μm. Finally, Ar-ion milling was performed to make them electron transparent. The Cu nanoparticles, after these procedures deposited by galvanic displacement on the Si surfaces, are ready for plan-view TEM measurements. For the TEM study, an FEI, Tecnai G^2^ F30, S-Twin microscope equipped with a Gatan Orius CCD camera was used. The microscope is equipped with a HAADF detector from Fischione (Model 3000) along with a scanning unit to perform the high-angle annular dark-field scanning transmission electron microscopy (STEM-HAADF). After the TEM analysis, the same samples were transferred to a rapid thermal annealing unit (model: JETFIRST100 jipelec) and annealing was performed in air, oxygen and nitrogen atmospheres, one by one. The rapid thermal annealing was performed at 500 °C for 1 min (a heating ramp rate of 10 °C/s) was used for all three different samples. Before and after the thermal annealing, the structural analysis was done using the selected area electron diffraction (SAED) in the TEM as well as by the X-ray diffraction (model: Rigaku TTRAX3, Cu Kα (λ = 1.5418 Å). To understand the interface and thermal evolution in different environments, cross-sectional TEM studies were also carried out. For preparing the cross-sectional samples, two substrates of 3 mm width were bonded face-to-face and then inserted into a brass tube. A 0.5 mm thick disk was cut from the tube and then mechanically thinned, dimpled, polished and finally ion-milled to make the sample electron transparent.

## Results and Discussion

**Figure 1:**
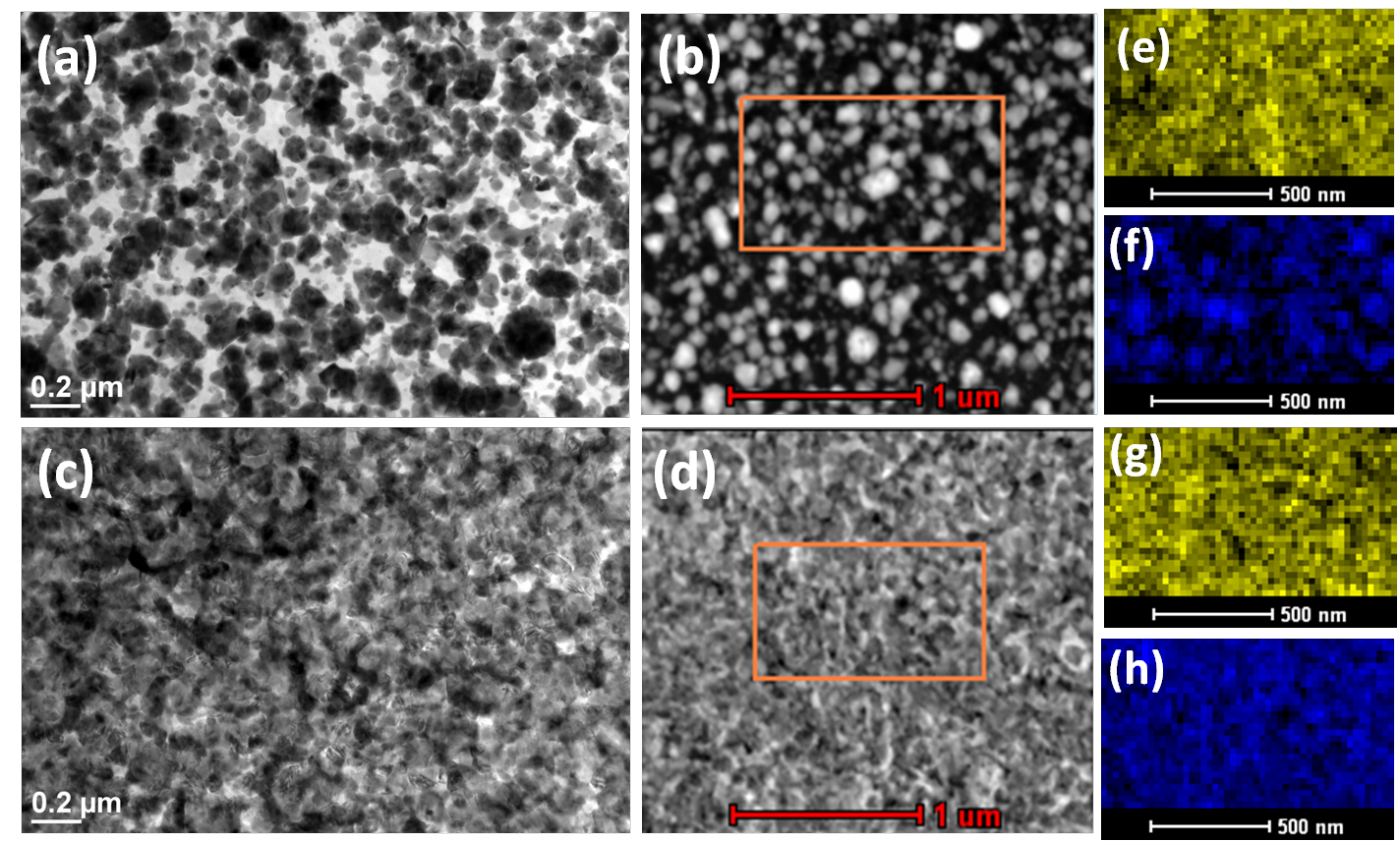
Plan view (a) TEM image (b) STEM-HAADF image of as-deposited copper nanoparticles on Si. Plan view (c) TEM image (d) STEM-HAADF image after the same nanoparticle substrate was annealed at 500 °C for 1 min under oxygen atmosphere. EDX elemental image (silicon (yellow) and copper (blue)): (e) and (f) collected from the orange rectangular area in figure (b); (g) and (h) collected from the orange rectangular area in figure (d).

Copper deposited on silicon surfaces by the galvanic displacement reaction were extensively examined by TEM. The plan-view bright field TEM image in Figure 1a and the STEM-HAADF image in Figure 1b show the deposited copper nanoparticles on a silicon substrate. The elemental composition is presented by the EDX mapping. Figure 1e and Figure 1f show the silicon (yellow) and copper (blue) elemental mapping, respectively, collected from a region marked by an orange rectangle in the STEM-HAADF image in Figure 1b. The thermal oxidation was performed in a rapid thermal annealing system at 500 °C for 1 min. We have analyzed the phase of the as-deposited material and the samples annealed under oxygen atmosphere by selected area electron diffraction (SAED).

**Figure 2:**
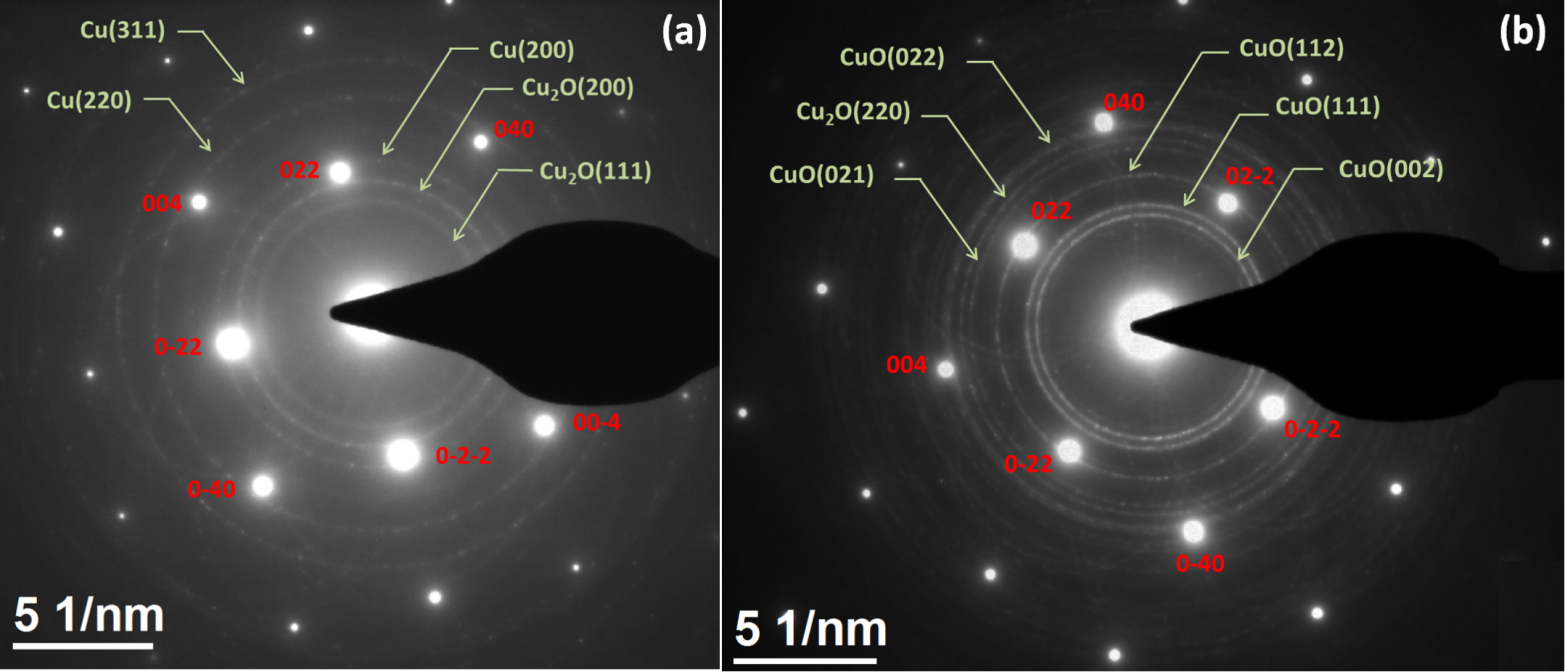
SAED pattern of (a) as-deposited copper nanoparticles on the silicon substrate (b) the same sample annealed at 500 °C in oxygen ambient. The bright spot (indexed in red) are from single crystal silicon and the ring patterns (indexed in green) are from polycrystalline Cu, Cu_2_O or CuO.

The diffraction pattern of the as-deposited sample (Figure 2a) shows some clear bright spots and faint rings. The bright spots were indexed as crystalline silicon in the [100] zone axis. The faint ring patterns correspond to the copper cubic structure along with the presence of Cu_2_O. The presence of Cu_2_O is due to oxidation of the Cu nanoparticle from air exposure. The diffraction pattern of the annealed sample at oxygen atmosphere shows bright ring patterns with the bright spots of crystalline silicon in [100] zone axis (Figure 2b). The bright ring pattern is indexed as the monoclinic (*a* = 4.682, *b* = 3.424, *c* = 5.127) CuO structure. The presence of Cu_2_O is also observed but there is no presence of Cu, indicating a complete oxidation.

**Figure 3:**
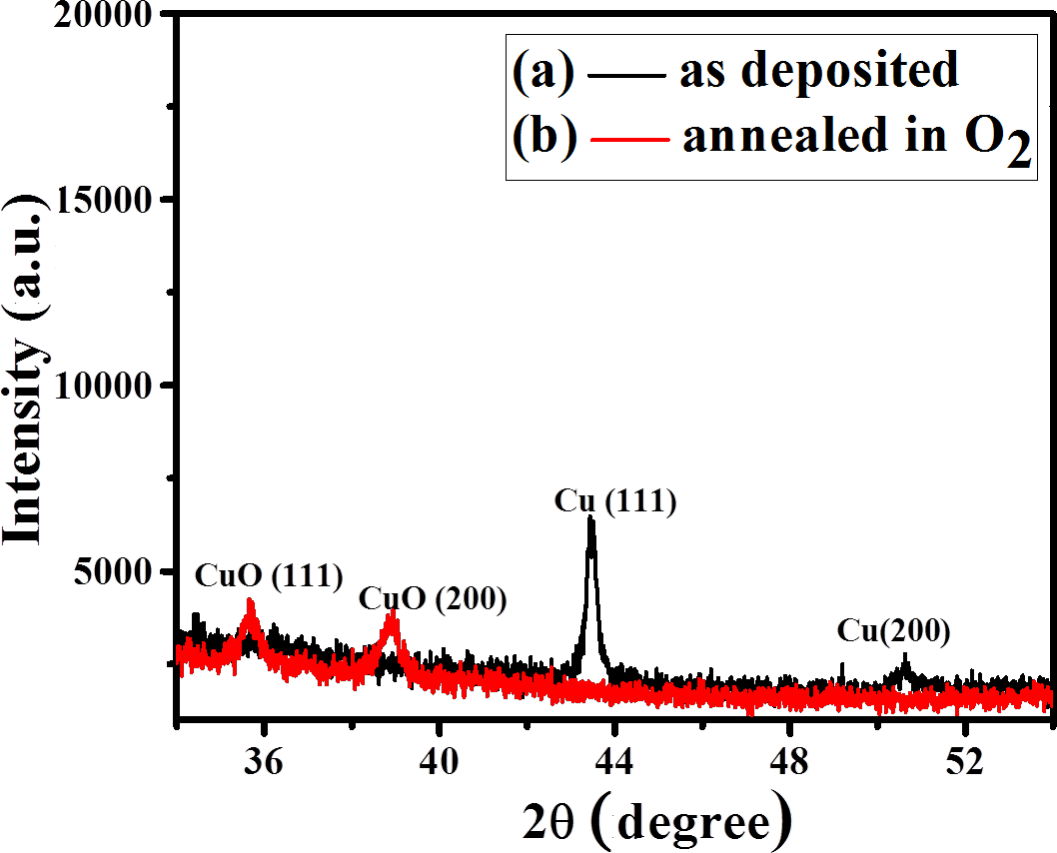
X-ray diffraction pattern for the (a) as-deposited copper nanoparticles on silicon substrate and (b) when the copper nanoparticle substrate is annealed under oxygen at 500 °C for 1 min.

The XRD pattern of the as-deposited Cu nanoparticles on the Si (Figure 3a) shows the peaks at 43.5° and 50.7° which correspond to the Cu(111) and Cu(200) planes [JCPDS No. 04-0836]. The XRD pattern of the annealed sample under oxygen atmosphere (Figure 3b) shows that the copper peak disappeared and the new peaks at 35.7° and 38.9° appear that correspond to the (111) and (200) planes of CuO, respectively [JCPDS No. 80-1916].

The thermal annealing of thin films on a substrate leads to the formation of islands, which is the consequence of the Gibbs–Thomson effect for the energy minimization [31]. There are different theoretical explanations regarding the mechanism of particle agglomeration. In general, a copper thin film is agglomerated via a grain boundary grooving mechanism during thermal annealing [32–33]. Further annealing results in Oswald ripening [34–35], where the atoms diffuse from smaller to larger nanoparticles and form larger copper clusters. The agglomeration of copper particles was observed in the earlier observations during thermal annealing either in vacuum or in inert ambient where the presence of oxygen was excluded. However, oxidation plays an important role in thermal evolution of the copper thin film. The formation of oxide impedes the agglomeration of copper film during thermal annealing [32]. It is also observed that when the substrate or the Cu film is exposed to air, the agglomeration is hindered [34]. Such retardation in copper agglomeration is caused as the oxidation in the grain boundaries retards the diffusion in the grain boundaries and also affects the grain boundary grooving [34,36–37]. Again the agglomeration has also been observed during the oxidation of copper nanoparticles on glass substrates [38]. Similar agglomeration has been found when cuprous oxide deposited on glass or copper and tin oxide-coated glass substrates, is annealed in oxygen to form CuO [29,39]. When Cu or Cu_2_O in powder form is annealed in an oxygen environment to form CuO, particle agglomeration is also observed [40]. Thus, in general, it can be said that the copper thin film tends to agglomerate during thermal annealing at high temperatures and oxidation has a strong influence.

We have studied the annealing of copper nanoparticles on silicon surfaces under various atmospheric conditions. The copper-containing substrate, annealed in an oxygen environment at 500 °C for 1 min, is presented in the TEM image in Figure 1c and STEM-HAADF image in Figure 1d (where the images of the as-deposited Cu nanoparticles on the Si surface are presented in Figure 1a and Figure 1b, respectively). We observed that for copper nanoparticles on silicon substrates annealed in an oxygen environment, the particles are wetted on the silicon surface. Figure 1g and Figure 1h present the silicon and the copper elemental mapping, respectively, collected from the orange square in the STEM-HAADF image of the annealed substrate (Figure 1d). If we compare Figure 1f (the as-deposited) and Figure 1h (annealed at oxygen), one can see the presence of elemental copper is discontinuous for the as-deposited substrate, whereas it is almost continuous when annealed in the oxygen environment. This supports the wetting on Si surfaces during the formation of CuO by the thermal oxidation.

**Figure 4:**
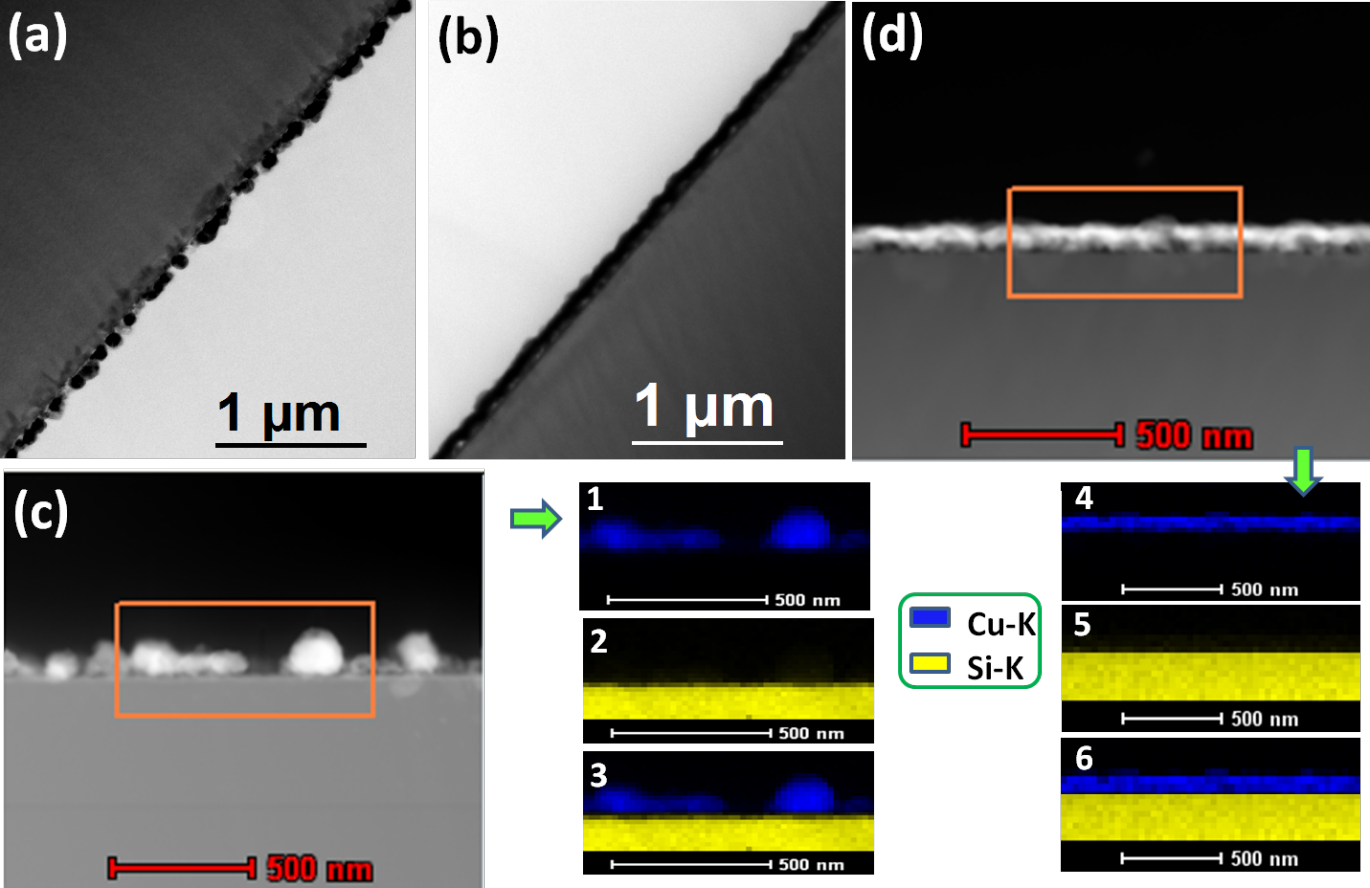
(a),(b) Cross-sectional bright field TEM images and (c),(d) STEM-HAADF images of as-deposited and annealed at 500 °C in oxygen ambient copper nanoparticles on silicon substrates, respectively. The elemental mapping of Cu (1 and 4), Si (2 and 5), and the composite thereof (3 and 6) collected from the orange rectangular region of STEM-HAADF images (c) and (d), respectively.

**Figure 5:**
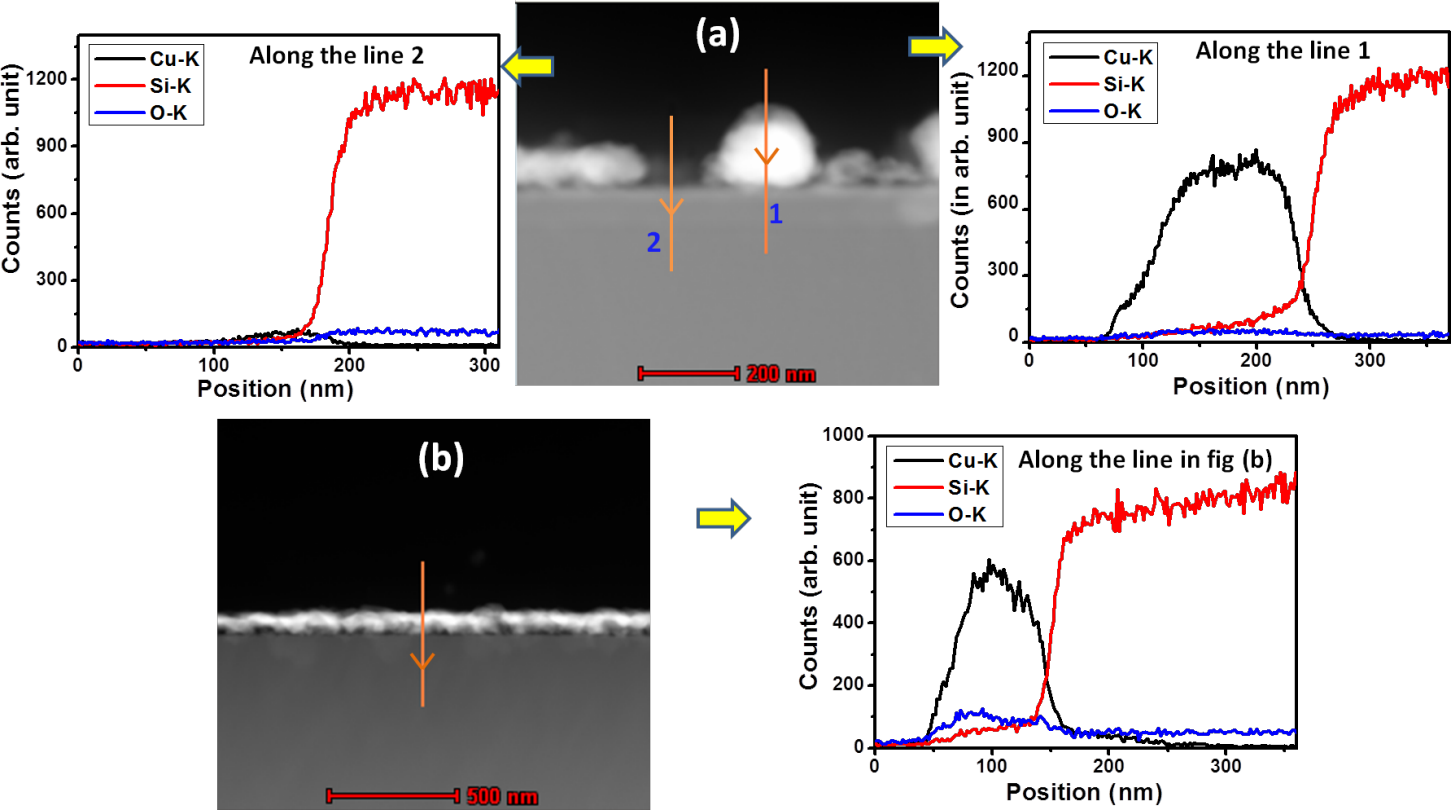
Copper, silicon and oxygen elemental line profiles collected from the region indicated by the arrows in the STEM-HAADF images of (a) as-deposited copper nanoparticles and (b) copper nanoparticles oxidized to CuO on the silicon substrate.

**Figure 6:**
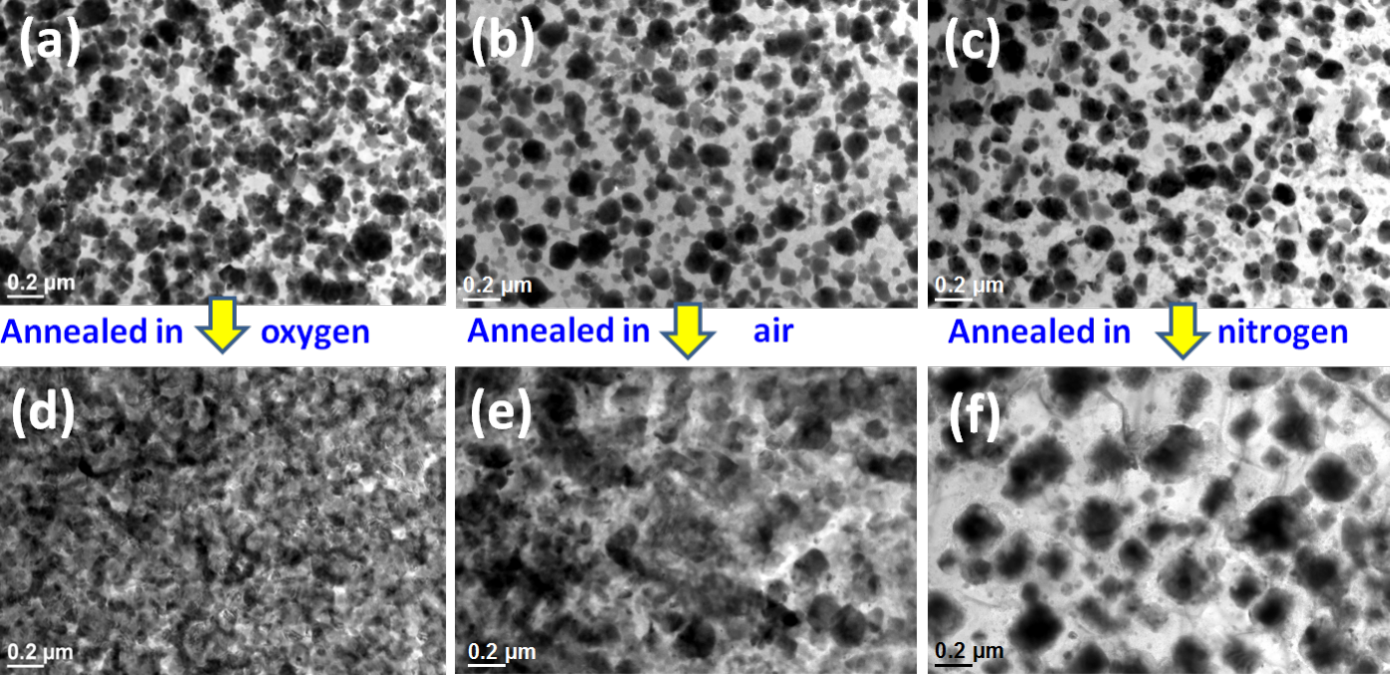
(a–c) Cu nanoparticles on Si substrates deposited via the galvanic displacement reaction, and (d–f) when the same substrates were annealed in oxygen, air, nitrogen, respectively.

The wetting during formation of CuO on the Si surface is more prominently visible from the cross-sectional TEM images. Figure 4a and Figure 4c are the cross-sectional TEM and STEM-HAADF images of the as-deposited Cu nanoparticles on the Si surface and Figure 4b and Figure 4d are the cross-sectional TEM and STEM-HAADF images when annealed in an oxygen environment at 500 °C for 1 minute. The as-deposited Cu nanoparticles are quiet distinct, where a continuous film is formed during the thermal oxidation of Cu. The elemental mapping of Cu (Figure 4-1 and Figure 4-4), Si (Figure 4-2 and Figure 4-5), and composite of them (Figure 4-3 and Figure 4-6) have been presented which were collected from the area outlined by the orange rectangle in Figure 4c and Figure 4d.

We have extended our study by measuring the elemental line profile using STEM-HAADF-EDX, as presented in Figure 5. Two elemental line profiles for Cu, O and Si have been acquired for the as-deposited sample (Figure 5a). These profiles show a higher Cu intensity when the electron beam (along the line) passes through the particle and significantly less intensity when it does not pass through any particle.

The elemental line profile for the same sample annealed in an oxygen environment at 500 °C for 1 minute shows high copper intensity as the electron beam passes through the film (Figure 5b). We have taken several such line scans on annealed samples and the result was the same all the cases. The higher oxygen intensity in the Cu film region also indicates the formation of CuO as compared to that found in the as-deposited sample.

A comparative study has been performed to observe the wetting behavior during the thermal annealing of Cu nanoparticles on Si surfaces at the same temperature (500 °C) and with the same annealing duration (1 min) but at different ambient conditions. Figure 6a–c reports on the results of as-deposited Cu nanoparticles on the silicon substrates deposited by the galvanic displacement reaction conducted for 2 min. Those same substrates are annealed under oxygen, air and nitrogen conditions and are presented in Figure 6d–f, respectively. One can clearly observe from the TEM images when the Cu particle-containing substrates are annealed under nitrogen atmosphere as agglomeration occurs, but when they are annealed under oxygen or in air, wetting occurs. The corresponding area coverage (in percentage) of the annealed substrate under different environmental conditions is presented in Table 1. Almost the complete area is covered (≈98%) due to wetting when the substrate is annealed in oxygen. We observed from the SAED and XRD analyses that in the process of annealing at 500 °C in oxygen, Cu particles were transformed into CuO. This is similar to the annealing of the Cu particles under the same time when annealing in air, which also leads to the formation of CuO (Supporting Information File 1, Figure S1).

**Table 1:** Area coverage for samples annealed under different ambient conditions.

	Percentage area coverage

As-deposited	63.8
Annealed in N_2_	43.3
Annealed in Air	92.4
Annealed in O_2_	97.6

**Figure 7:**
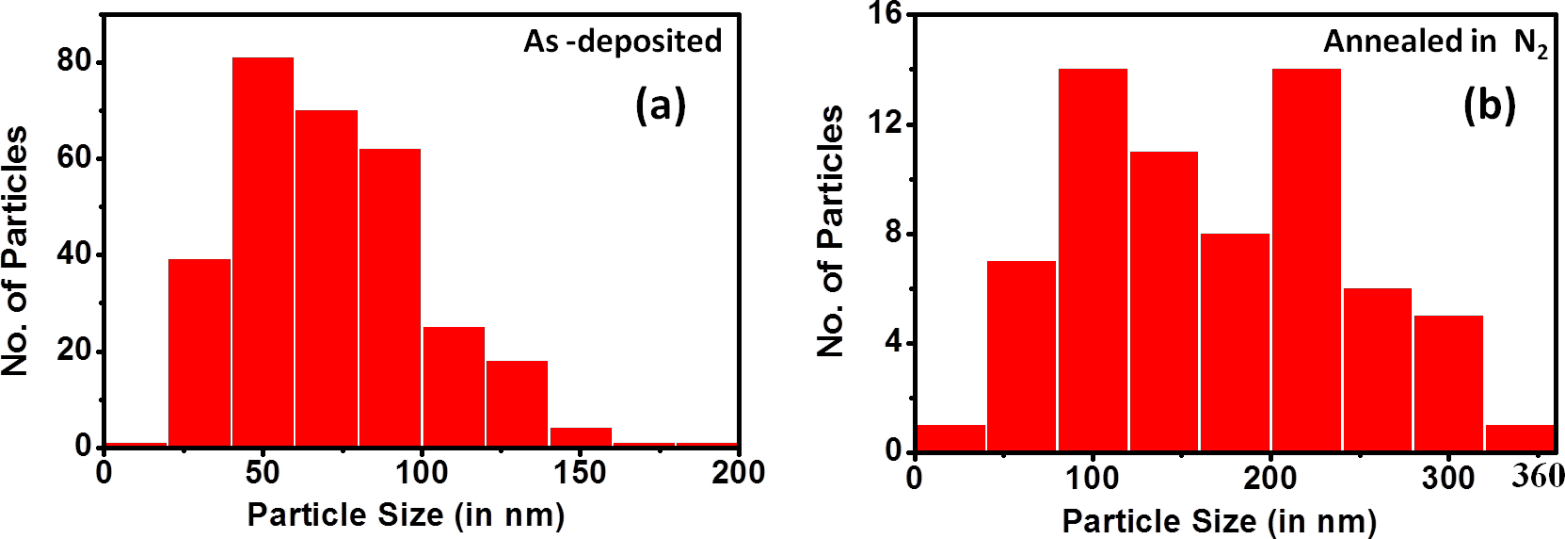
Cu nanoparticle distribution on a Si surface for (a) as-deposited and (b) annealed in nitrogen samples. A summary of the distributions is presented in Table 2.

**Figure 8:**
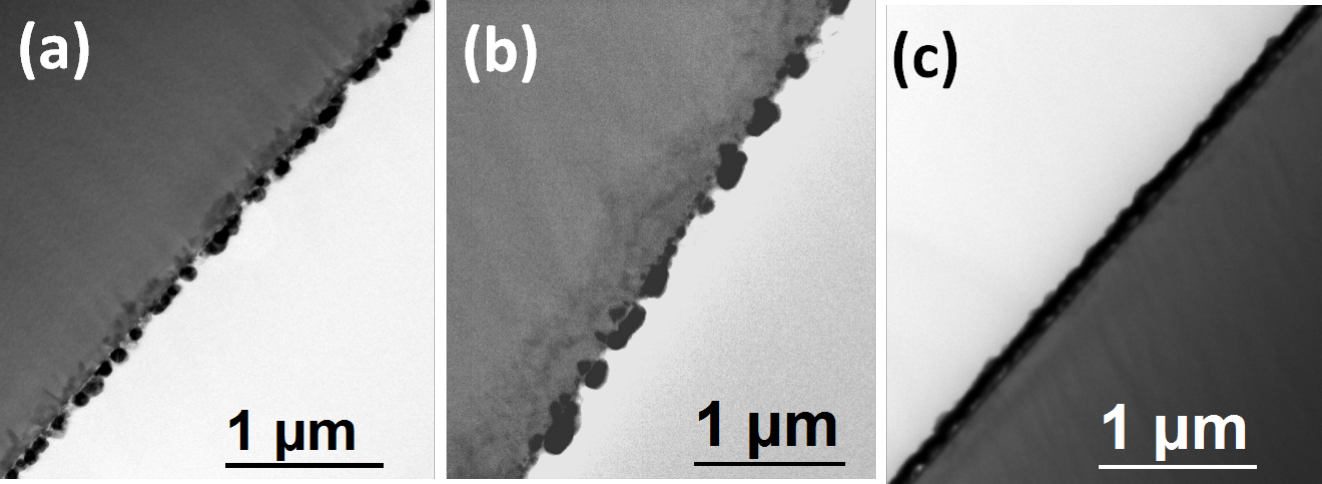
(a) As-deposited Cu nanoparticles on Si and when annealed at 500 °C for 1 min in (b) nitrogen and (c) oxygen.

We observed wetting under both the circumstances. In contrast, when annealing in nitrogen atmosphere, we observe agglomeration. The particle distribution is shown in Figure 7 and the summary is presented in the Table 2. For the samples annealed in the nitrogen environment, the Cu phase remains unchanged (Supporting Information File 1, Figure S2). This is consistent with the observation of Li et al. [35] that the annealing of Cu nanoparticles in a nitrogen environment does not change the Cu crystallinity. During the formation of copper silicide, agglomeration also occurs [41]. Thus the oxidation of copper nanoparticles on silicon leads to wetting rather than agglomeration. The cross-sectional TEM images of the as-deposited and annealed under nitrogen and oxygen are presented in the Figure 8. We observe larger particle formation due to the agglomeration when annealed in a nitrogen environment and a continuous film formation due to wetting when annealed in an oxygen environment.

**Table 2:** Summary of the particle distribution presented in Figure 7.

	As-deposited	Annealed in N_2_

Number of particles	303	67
Mean diameter (nm)	72.4	167.6
Standard deviation (nm)	31.3	74.9

The wetting phenomena of nanoparticles on a substrate surface is a consequence of the negative interface energy between the particles and the substrate. This was reported on by Yang et al. in their study on platinum nanocrystals on silica surfaces [42]. In another study it was observed that when copper nanoparticles are deposited on silicon surfaces and oxidized at high temperatures to form CuO, the shape of the CuO particle appeared lamellar [43]. The thermal annealing in the oxygen environment may also produce SiO_2_ at the silicon–copper surface that may affect the interface energy, but as the Cu particles are spread all over the surface, the oxygen exposure of the Si surface is reduced. Again, when the copper film is oxidized to form CuO, the film thickness is increased; such a thickness increment is due to the lattice expansion as the cubic copper lattice is transformed into a monoclinic CuO structure [3,44]. On the other hand, the surface energy of the CuO thin film is greater than that of the Cu_2_O thin film [45]. Thus combining the lattice expansion and the surface energy increment, wetting takes place during formation of CuO on the silicon surface. The wetting which occurs during the formation of CuO suggests a strong interaction between the CuO and SiO_2_/Si surfaces.

## Conclusion

In this work, we investigate the role of oxygen in wetting Cu nanoparticles on silicon (100) substrates deposited by the galvanic displacement reaction using XRD, TEM and EDX. The rapid thermal annealing in oxygen atmosphere at 500 °C shows the formation of monoclinic CuO. Annealing under a nitrogen environment does not convert the copper to any other phase. Annealing in nitrogen leads to agglomeration due to the surface energy minimization. The oxidation of the copper nanoparticles on the silicon surface by rapid thermal annealing shows that during the oxidation wetting occurs rather than agglomeration. The lattice transformation as well as the surface energy modification during the CuO formation by the thermal oxidation of Cu nanoparticles on the silicon surface leads to the wetting. Such wetting implies a strong interaction between the CuO and the SiO_2_/Si surfaces. This study may be useful to understand and improve the efficiency of p-CuO/n-Si heterojunction solar cells.

## Supporting Information

File 1 Additional experimental data.
