# Supplementary material for: Role of oxygen in wetting of copper nanoparticles on silicon surfaces at elevated temperature
Source: Beilstein J Nanotechnol. 2017 Feb 13;8:425–33. doi: 10.3762/bjnano.8.45 (PMC5331294; doi:10.3762/bjnano.8.45)
Supplement: File 1 — Additional experimental data. [file Beilstein_J_Nanotechnol-08-425-s001.pdf]

Supporting Information

for

Role of oxygen in wetting of copper nanoparticles on silicon surfaces at elevated temperature

Tapas Ghosh and Biswarup Satpati*

Address: Surface Physics and Material Science Division, Saha Institute of Nuclear Physics, HBNI, 1/AF Bidhannagar, Kolkata-700064, India

Email: Biswarup Satpati - biswarup.satpati@saha.ac.in

* Corresponding author

Additional experimental data

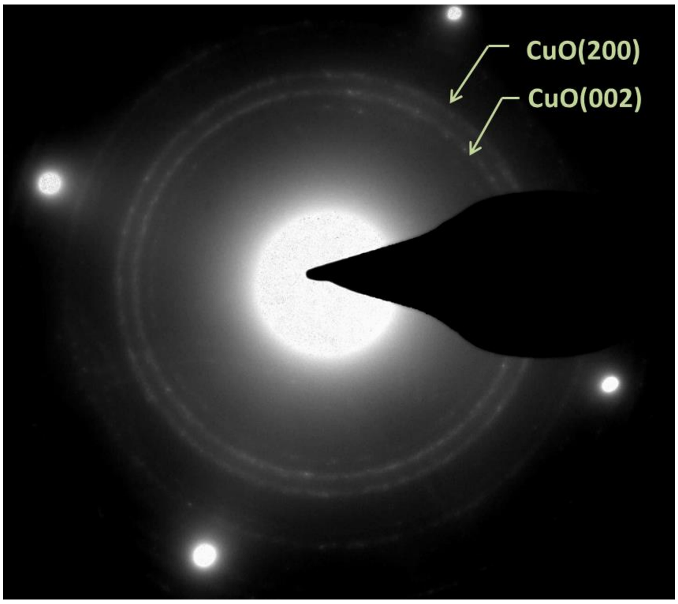

Figure S1: The selected area electron diffraction (SAED) of air annealed sample. The ring diffraction pattern shows the formation of CuO.

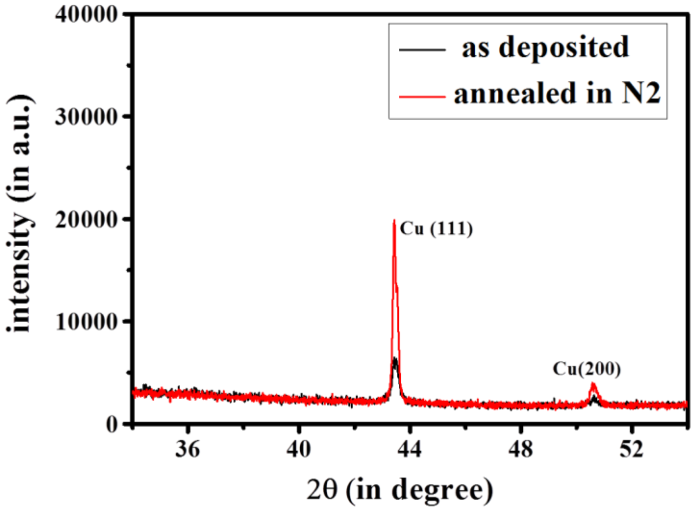

Figure S2: XRD patterns of the as deposited and the N_2 environment annealed samples. It is visible that the crystallinity of the Cu nanoparticles remains unchanged.
